# Supplementary material for: A Likelihood Approach for Real-Time Calibration of Stochastic Compartmental Epidemic Models
Source: PLoS Comput Biol. 2017 Jan 17;13(1):e1005257. doi: 10.1371/journal.pcbi.1005257 (PMC5240920; doi:10.1371/journal.pcbi.1005257)
Supplement: S1 File — (TAR.GZ) [file pcbi.1005257.s014.tar.gz › HSPH_Online-SI-Revision/Additions/PF09/n100-extreme-SEITR/n100-extreme-SEITR_PF_table8.pdf]

1	R0	{1.38654, 9.85565}	{2.19999, 20.}	{{2.19315, 20.}, {2.20361, 20.}}	{58.6678, 102.929}	inf	78.	9130.46	{9101.89, 9152.55}	1287.61	1.14227	newly	382.	2931.02	{2863., 2990.}	667.283	0.8849	Reff	0.108483	0.512182	{0.509582, 0.51503}	372.132	ar	92 379.	91 971.1	{91 602., 92 249.}	0.44653
2	R0	{2.36629, 12.2029}	{3., 20.}	{{3., 20.}, {3., 20.}}	{26.7807, 63.8955}	inf	5.	8476.73	{8465.11, 8489.96}	1130.29	1.09001	newly	300.	2595.08	{2538., 2650.}	765.027	0.936966	Reff	0.0235682	0.220023	{0.219061, 0.221067}	833.556	ar	99 022.	101 287.	{100 975., 101 619.}	2.28757
3	R0	{2.32253, 12.836}	{3., 20.}	{{3., 20.}, {3., 20.}}	{29.1695, 55.8118}	inf	20.	9121.39	{9109.98, 9138.12}	758.08	0.933528	newly	463.	2796.33	{2750., 2842.}	503.959	0.780963	Reff	0.0245491	0.23214	{0.231018, 0.233151}	845.615	ar	98 997.	100 794.	{100 365., 101 142.}	1.81485
4	R0	{1.79075, 11.2058}	{3., 20.}	{{3., 20.}, {3., 20.}}	{67.5276, 78.479}	inf	31.	7181.89	{7149.17, 7222.05}	767.379	0.938205	newly	412.	2191.55	{2141., 2244.}	431.93	0.725788	Reff	0.0574114	0.207592	{0.204504, 0.209792}	261.586	ar	96 920.	104 279.	{103 587., 104 888.}	7.5932
5	R0	{2.26952, 9.62371}	{3., 20.}	{{3., 20.}, {3., 20.}}	{32.1865, 107.82}	inf	0.	9395.93	{9388.55, 9403.79}	4924.56	1.7011	newly	78.	2879.01	{2818., 2940.}	3591.04	1.56709	Reff	0.0257137	0.231912	{0.231011, 0.233443}	801.902	ar	98 881.	101 699.	{101 422., 102 076.}	2.85023
6	R0	{1.57191, 6.89004}	{3., 20.}	{{3., 20.}, {3., 20.}}	{90.8506, 190.274}	inf	5.	7965.7	{7953.59, 7977.98}	14 651.3	2.16883	newly	40.	2441.99	{2387., 2496.}	6004.98	1.78562	Reff	0.077951	0.215676	{0.21496, 0.21671}	176.682	ar	95 046.	104 087.	{103 701., 104 501.}	9.51239
7	R0	{1.99226, 17.2339}	{2.12573, 20.}	{{2.1239, 20.}, {2.12734, 20.}}	{6.69932, 16.0503}	inf	78.	9226.12	{9209.35, 9238.}	222.029	0.507894	newly	982.	2958.75	{2893., 3018.}	201.298	0.478941	Reff	0.0456427	0.493463	{0.49226, 0.494565}	981.143	ar	97 992.	90 035.6	{89 809., 90 269.}	8.11939
8	R0	{2.63369, 15.1879}	{3., 20.}	{{3., 20.}, {3., 20.}}	{13.9086, 31.6838}	inf	5.	7841.12	{7833.02, 7851.88}	449.098	0.73965	newly	510.	2397.66	{2342., 2451.}	370.129	0.672154	Reff	0.0154861	0.217462	{0.216831, 0.218162}	1304.24	ar	99 451.	101 262.	{100 968., 101 494.}	1.82087
9	R0	{2.53271, 9.90552}	{3., 20.}	{{3., 20.}, {3., 20.}}	{18.4502, 101.908}	inf	2.	11 285.5	{11 277.4, 11 294.6}	6499.69	1.81952	newly	96.	3473.61	{3402., 3541.}	3518.34	1.55846	Reff	0.0167159	0.25318	{0.251721, 0.254661}	1414.61	ar	99 355.	101 387.	{101 052., 101 707.}	2.04538
10	R0	{2.48791, 18.9003}	{2.79993, 20.}	{{2.78469, 20.}, {2.84756, 20.}}	{12.5415, 5.81843}	inf	42.	7887.66	{7783.12, 7929.04}	109.722	0.321634	newly	1167.	2436.79	{2371., 2501.}	108.808	0.319654	Reff	0.0212716	0.270609	{0.264058, 0.27295}	1172.16	ar	99 291.	97 548.6	{97 091., 98 853.}	1.75486
11	R0	{2.84414, 18.4765}	{2.2671, 20.}	{{2.26289, 20.}, {2.26975, 20.}}	{20.2888, 8.24561}	inf	13.	13 604.1	{13 583.1, 13 624.7}	333.113	0.636601	newly	1003.	4381.95	{4292., 4457.}	336.884	0.640327	Reff	0.0117463	0.521022	{0.518889, 0.52337}	4335.63	ar	99 654.	92 853.8	{92 565., 93 125.}	6.82383
12	R0	{2.29369, 14.622}	{2.92128, 20.}	{{2.91755, 20.}, {2.92383, 20.}}	{27.3617, 36.7802}	inf	14.	7494.9	{7484.68, 7508.74}	449.077	0.739633	newly	542.	2300.77	{2250., 2347.}	324.496	0.627809	Reff	0.0247948	0.248308	{0.247788, 0.249147}	901.452	ar	98 982.	98 769.1	{98 654., 98 869.}	0.21509
13	R0	{2.73413, 16.7529}	{2.80486, 20.}	{{2.80302, 20.}, {2.80658, 20.}}	{2.58693, 19.3823}	inf	10.	9249.2	{9240.38, 9258.33}	351.181	0.65435	newly	706.	2861.92	{2811., 2916.}	305.371	0.607808	Reff	0.0138347	0.28387	{0.283186, 0.284522}	1951.87	ar	99 543.	97 203.1	{97 093., 97 303.}	2.35068
14	R0	{1.79554, 16.5943}	{1.93854, 20.}	{{1.936, 20.}, {1.94226, 20.}}	{7.96426, 20.5233}	inf	185.	11 026.6	{11 015., 11 043.6}	191.94	0.465291	newly	1313.	3625.	{3570., 3701.}	176.085	0.440999	Reff	0.0662734	0.658719	{0.656387, 0.660387}	893.942	ar	96 821.	83 556.2	{83 323., 83 846.}	13.7004
15	R0	{1.91429, 13.7771}	{1.88971, 20.}	{{1.88515, 20.}, {1.88954, 20.}}	{1.33539, 45.1684}	inf	37.	12 057.6	{12 021.2, 12 059.1}	720.806	0.914233	newly	621.	4037.86	{3959., 4112.}	550.219	0.813009	Reff	0.0501544	0.756098	{0.756112, 0.759033}	1407.54	ar	97 541.	81 403.5	{81 038., 81 685.}	16.5444
16	R0	{1.42094, 10.5871}	{2.15857, 17.197}	{{2.1573, 17.1913}, {2.16223, 17.2446}}	{51.9114, 62.4333}	inf	126.	7589.04	{7562.99, 7636.31}	531.894	0.800624	newly	644.	2762.34	{2702., 2816.}	328.935	0.63233	Reff	0.103388	0.454849	{0.453434, 0.456229}	339.945	ar	93 044.	87 786.8	{87 674., 87 993.}	5.65019
17	R0	{1.45515, 6.16466}	{3., 20.}	{{3., 20.}, {3., 20.}}	{106.164, 224.43}	inf	5.	9401.79	{9388.99, 9415.48}	18 703.6	2.27424	newly	41.	2872.57	{2813., 2932.}	6906.27	1.84543	Reff	0.0982954	0.225941	{0.224636, 0.226835}	129.859	ar	93 254.	103 577.	{103 063., 103 960.}	11.0701
18	R0	{2.85669, 13.2416}	{3., 20.}	{{3., 20.}, {3., 20.}}	{5.01665, 51.0391}	inf	1.	9515.97	{9503.11, 9530.17}	1123.13	1.08747	newly	314.	2917.25	{2859., 2970.}	829.061	0.967997	Reff	0.0106269	0.23678	{0.235563, 0.238014}	2128.12	ar	99 644.	101 759.	{101 442., 102 048.}	2.12287
19	R0	{2.56417, 17.9558}	{2.24008, 20.}	{{2.23882, 20.}, {2.24116, 20.}}	{12.639, 11.3846}	inf	35.	12 634.	{12 625.8, 12 643.9}	294.443	0.595984	newly	1136.	4055.78	{3979., 4128.}	257.023	0.552656	Reff	0.01818	0.510693	{0.509846, 0.511557}	2709.1	ar	99 389.	91 684.9	{91 495., 91 855.}	7.75142
20	R0	{2.27517, 11.1864}	{3., 20.}	{{3., 20.}, {3., 20.}}	{31.8583, 78.7885}	inf	9.	8099.06	{8075.99, 8118.98}	1660.66	1.24568	newly	219.	2475.45	{2412., 2529.}	1030.34	1.05314	Reff	0.0255502	0.214221	{0.212614, 0.215453}	738.434	ar	98 894.	104 175.	{103 738., 104 673.}	5.33957
21	R0	{2.35735, 10.4735}	{3., 20.}	{{3., 20.}, {3., 20.}}	{27.2615, 90.9581}	inf	3.	9755.17	{9742.82, 9770.23}	3162.6	1.51356	newly	156.	2987.31	{2924., 3054.}	1814.94	1.2821	Reff	0.0230549	0.231872	{0.230454, 0.233134}	905.737	ar	99 036.	102 495.	{102 149., 102 894.}	3.49277
22	R0	{1.79551, 11.9179}	{2.99967, 20.}	{{3., 20.}, {3., 20.}}	{67.0651, 67.8148}	inf	31.	6503.69	{6486.58, 6529.29}	634.051	0.865724	newly	400.	1990.93	{1942., 2037.}	397.733	0.696929	Reff	0.0555172	0.218205	{0.215587, 0.219543}	293.041	ar	97 033.	101 091.	{100 246., 101 576.}	4.18246
23	R0	{1.23545, 6.31687}	{3., 20.}	{{3., 20.}, {3., 20.}}	{142.827, 216.612}	inf	19.	5959.13	{5951.87, 5969.12}	5919.32	1.77955	newly	79.	1821.59	{1768., 1865.}	2205.81	1.36273	Reff	0.139655	0.206768	{0.206059, 0.207649}	48.0562	ar	88 739.	100 007.	{99 812., 100 208.}	12.6981
24	R0	{2.24089, 10.1789}	{3., 20.}	{{3., 20.}, {3., 20.}}	{33.8754, 96.4849}	inf	7.	10 471.9	{10 460.8, 10 481.}	2917.84	1.4797	newly	171.	3219.4	{3148., 3294.}	1782.69	1.27472	Reff	0.025703	0.242691	{0.241833, 0.24355}	844.214	ar	98 868.	100 865.	{100 482., 101 139.}	2.02032
25	R0	{1.57298, 10.2607}	{2.17273, 16.6125}	{{2.17044, 16.5871}, {2.17402, 16.6417}}	{38.1284, 61.9042}	inf	47.	8349.7	{8308.83, 8389.74}	859.736	0.982147	newly	443.	3142.56	{3078., 3202.}	609.381	0.850833	Reff	0.0819837	0.465886	{0.46491, 0.46795}	468.267	ar	94 957.	87 435.2	{87 280., 87 586.}	7.92131
26	R0	{1.2154, 8.2576}	{2.15736, 16.9628}	{{2.15531, 16.9237}, {2.15909, 16.9957}}	{77.5021, 105.421}	inf	88.	6195.83	{6156.1, 6232.11}	1080.16	1.07194	newly	345.	2272.08	{2212., 2327.}	558.574	0.818528	Reff	0.143952	0.427816	{0.426998, 0.428679}	197.194	ar	88 421.	88 939.3	{88 772., 89 086.}	0.586162
27	R0	{2.22841, 18.7983}	{2.17611, 17.0254}	{{2.17314, 16.9829}, {2.17822, 17.0719}}	{2.347, 9.43097}	inf	67.	6591.24	{6532.09, 6655.81}	88.9691	0.276376	newly	1079.	2409.91	{2349., 2465.}	123.347	0.348908	Reff	0.0322005	0.419483	{0.417992, 0.421012}	1202.72	ar	98 756.	91 729.6	{91 554., 91 919.}	7.11488
28	R0	{1.27778, 5.36767}	{3., 20.}	{{3., 20.}, {3., 20.}}	{134.782, 272.601}	inf	7.	8472.7	{8461.9, 8482.29}	24 819.7	2.39654	newly	32.	2594.09	{2539., 2658.}	8006.53	1.90877	Reff	0.130065	0.223894	{0.222985, 0.22506}	72.14	ar	89 843.	101 478.	{101 103., 101 794.}	12.9503
29	R0	{2.38042, 15.8901}	{3., 20.}	{{3., 20.}, {3., 20.}}	{26.0282, 25.8645}	inf	37.	7894.62	{7883.32, 7911.3}	239.554	0.530908	newly	849.	2414.53	{2361., 2465.}	184.397	0.453851	Reff	0.0237804	0.229554	{0.22893, 0.230228}	865.307	ar	99 112.	97 957.5	{97 808., 98 101.}	1.16485
30	R0	{2.53188, 12.7607}	{3., 20.}	{{3., 20.}, {3., 20.}}	{18.489, 56.7312}	inf	11.	10 534.1	{10 514.3, 10 549.5}	950.263	1.0213	newly	428.	3232.15	{3167., 3299.}	655.175	0.877994	Reff	0.0182295	0.237492	{0.236261, 0.238921}	1202.79	ar	99 317.	102 993.	{102 592., 103 361.}	3.70107
31	R0	{1.24482, 10.5471}	{2.28831, 20.}	{{2.28691, 20.}, {2.2893, 20.}}	{83.8262, 89.6256}	inf	170.	6467.08	{6453.25, 6484.28}	405.241	0.703497	newly	689.	2027.82	{1977., 2067.}	194.313	0.468749	Reff	0.142009	0.370673	{0.369806, 0.371848}	161.02	ar	89 097.	96 430.3	{96 222., 96 644.}	8.23068
32	R0	{2.30446, 17.9607}	{3., 20.}	{{3., 20.}, {3., 20.}}	{30.1823, 11.3542}	inf	48.	6139.26	{6116.13, 6163.98}	82.5532	0.261387	newly	1094.	1876.38	{1832., 1916.}	71.5155	0.234228	Reff	0.0266626	0.213142	{0.212266, 0.214091}	699.404	ar	99 028.	98 448.8	{98 250., 98 624.}	0.584845
33	R0	{2.31579, 11.8236}	{3., 20.}	{{3., 20.}, {3., 20.}}	{29.5454, 69.1532}	inf	5.	7506.05	{7489.96, 7520.46}	1473.6	1.19689	newly	197.	2286.4	{2234., 2340.}	1060.61	1.06463	Reff	0.0234821	0.20522	{0.203735, 0.206888}	773.942	ar	99 012.	105 118.	{104 575., 105 511.}	6.16643
34	R0	{1.6678, 10.6744}	{3., 20.}	{{3., 20.}, {3., 20.}}	{79.8777, 87.3642}	inf	36.	5712.99	{5699.31, 5726.55}	898.774	0.999467	newly	278.	1746.37	{1703., 1790.}	528.191	0.798011	Reff	0.0712985	0.209236	{0.208645, 0.209855}	193.466	ar	95 809.	98 967.1	{98 807., 99 147.}	3.29619
35	R0	{1.65476, 7.07559}	{3., 20.}	{{3., 20.}, {3., 20.}}	{81.2952, 182.662}	inf	5.	9540.05	{9534.07, 9546.07}	13 726.2	2.1407	newly	50.	2927.23	{2863., 2987.}	5754.46	1.76742	Reff	0.0705259	0.232785	{0.231727, 0.233961}	230.07	ar	95 746.	102 559.	{102 164., 102 838.}	7.11604
36	R0	{2.71807, 18.5959}	{2.94683, 20.}	{{2.93695, 20.}, {2.95254, 20.}}	{8.4162, 7.55059}	inf	24.	8400.52	{8379.28, 8434.23}	122.472	0.347273	newly	1211.	2581.2	{2526., 2631.}	113.146	0.328624	Reff	0.0154386	0.238579	{0.236872, 0.240464}	1445.34	ar	99 540.	99 851.1	{99 498., 100 077.}	0.331123
37	R0	{2.6202, 12.7552}	{2.86569, 20.}	{{2.86354, 20.}, {2.86744, 20.}}	{9.36902, 56.7988}	inf	9.	11 694.4	{11 685.2, 11 703.2}	1239.57	1.12696	newly	383.	3617.11	{3545., 3680.}	844.415	0.975115	Reff	0.016088	0.294341	{0.293427, 0.296054}	1729.56	ar	99 426.	99 031.3	{98 806., 99 271.}	0.397009
38	R0	{1.47672, 9.81651}	{2.14363, 16.3753}	{{2.13758, 16.2087}, {2.14817, 16.5697}}	{45.1619, 66.8143}	inf	40.	6816.85	{6668.53, 6982.78}	1098.04	1.07837	newly	323.	2585.71	{2518., 2666.}	700.529	0.903278	Reff	0.0934468	0.44878	{0.444869, 0.453211}	380.252	ar	93 815.	87 328.3	{87 137., 87 484.}	6.91433
39	R0	{1.90004, 12.3313}	{2.87604, 20.}	{{2.87222, 20.}, {2.87884, 20.}}	{51.3672, 62.1889}	inf	30.	7426.91	{7414.49, 7443.35}	601.314	0.845912	newly	464.	2285.33	{2230., 2335.}	392.528	0.692353	Reff	0.0517191	0.254423	{0.253591, 0.255244}	391.932	ar	97 413.	99 066.1	{98 912., 99 205.}	1.69701
40	R0	{2.64218, 18.5237}	{3., 20.}	{{3., 20.}, {3., 20.}}	{13.5426, 7.96979}	inf	25.	6023.39	{6005.98, 6034.23}	116.825	0.336108	newly	874.	1839.5	{1797., 1884.}	110.469	0.323111	Reff	0.0162494	0.214014	{0.213323, 0.214863}	1217.06	ar	99 474.	100 378.	{100 131., 100 549.}	0.909132
41	R0	{1.6945, 7.17996}	{3., 20.}	{{3., 20.}, {3., 20.}}	{77.0434, 178.553}	inf	4.	10 653.1	{10 640.3, 10 669.5}	10 242.8	2.01464	newly	74.	3279.28	{3228., 3338.}	4331.46	1.6465	Reff	0.0626626	0.249324	{0.248033, 0.250423}	297.884	ar	96 332.	101 077.	{100 725., 101 415.}	4.92561
42	R0	{1.84559, 18.0074}	{2.28269, 20.}	{{2.27347, 20.}, {2.29052, 20.}}	{23.6835, 11.0655}	inf	180.	7738.57	{7709.31, 7767.55}	94.7791	0.28954	newly	1374.	2436.16	{2388., 2481.}	77.3042	0.248667	Reff	0.0586713	0.394083	{0.390917, 0.39718}	571.679	ar	97 358.	96 303.1	{95 660., 96 852.}	1.0835
43	R0	{2.24965, 17.0361}	{2.88497, 20.}	{{2.87929, 20.}, {2.88919, 20.}}	{28.2407, 17.3978}	inf	59.	7450.22	{7422.45, 7482.46}	138.56	0.377595	newly	1018.	2294.65	{2240., 2351.}	125.408	0.352886	Reff	0.0319	0.253702	{0.252595, 0.254775}	695.302	ar	98 770.	99 310.6	{99 180., 99 438.}	0.547363
44	R0	{2.54324, 15.7967}	{3., 20.}	{{3., 20.}, {3., 20.}}	{17.9598, 26.6087}	inf	13.	7251.63	{7247.32, 7257.53}	346.529	0.64985	newly	626.	2214.61	{2172., 2259.}	253.772	0.548672	Reff	0.0177772	0.220419	{0.219848, 0.221181}	1139.9	ar	99 354.	99 836.2	{99 619., 100 050.}	0.485325
45	R0	{2.54943, 14.8032}	{3., 20.}	{{3., 20.}, {3., 20.}}	{17.6734, 35.1059}	inf	8.	7713.25	{7700.73, 7725.17}	465.487	0.752423	newly	536.	2357.91	{2309., 2407.}	339.909	0.643304	Reff	0.0170047	0.216564	{0.215699, 0.217609}	1173.55	ar	99 380.	100 999.	{100 665., 101 317.}	1.62903
46	R0	{2.37755, 12.6461}	{3., 20.}	{{3., 20.}, {3., 20.}}	{26.1803, 58.1515}	inf	9.	9051.06	{9038.91, 9065.72}	914.692	1.00633	newly	377.	2772.99	{2706., 2839.}	635.541	0.866543	Reff	0.0224678	0.231452	{0.230596, 0.232668}	930.147	ar	99 096.	100 428.	{100 105., 100 756.}	1.34401
47	R0	{1.69279, 11.6636}	{2.23838, 17.5526}	{{2.22642, 17.5235}, {2.24421, 17.5799}}	{32.2305, 50.4904}	inf	61.	9389.02	{9338.97, 9437.61}	699.746	0.902948	newly	550.	3363.74	{3299., 3421.}	511.589	0.786414	Reff	0.0676608	0.455991	{0.453439, 0.460659}	573.936	ar	96 214.	90 019.4	{89 841., 90 204.}	6.43831
48	R0	{1.82402, 10.0038}	{3., 20.}	{{3., 20.}, {3., 20.}}	{64.4719, 99.924}	inf	27.	8354.76	{8344.25, 8365.3}	1594.68	1.22909	newly	248.	2559.15	{2495., 2609.}	931.915	1.01358	Reff	0.054447	0.219761	{0.218811, 0.220772}	303.624	ar	97 076.	101 876.	{101 602., 102 152.}	4.94502
49	R0	{1.6424, 11.1667}	{3., 20.}	{{3., 20.}, {3., 20.}}	{82.6595, 79.1039}	inf	29.	5106.98	{5091.63, 5125.47}	659.967	0.880793	newly	321.	1559.23	{1516., 1599.}	385.741	0.686315	Reff	0.0752383	0.19865	{0.197655, 0.1998}	164.027	ar	95 546.	100 389.	{100 023., 100 680.}	5.06854
50	R0	{2.05638, 14.3048}	{3., 20.}	{{3., 20.}, {3., 20.}}	{45.8874, 39.8132}	inf	38.	6414.66	{6397.38, 6445.15}	303.691	0.606048	newly	636.	1955.43	{1903., 1998.}	207.458	0.487708	Reff	0.0397498	0.208661	{0.20776, 0.209527}	424.937	ar	98 194.	100 606.	{100 375., 100 829.}	2.45673
